# New Developments in the Treatment of Schizophrenia: An Expert Roundtable

**DOI:** 10.1093/ijnp/pyad011

**Published:** 2023-03-18

**Authors:** Joshua T Kantrowitz, Christoph U Correll, Rakesh Jain, Andrew J Cutler

**Affiliations:** Area Psychosis, New York State Psychiatric Institute, New York, New York, USA; Schizophrenia Research, Nathan Kline Institute, Orangeburg, New York, USA; Department of Psychiatry, Columbia University College of Physicians and Surgeons, New York, New York, USA; Department of Psychiatry Research, The Zucker Hillside Hospital, Glen Oaks, New York, USA; Department of Psychiatry and Molecular Medicine, Donald and Barbara Zucker School of Medicine at Hofstra/Northwell, Hempstead, New York, USA; Department of Child and Adolescent Psychiatry, Charité Universitätsmedizin Berlin, Berlin, Germany; Department of Psychiatry, Texas Tech University School of Medicine-Permian Basin, Midland, Texas, USA; Department of Psychiatry, SUNY Upstate Medical University, Lakewood Ranch, Florida, USA

**Keywords:** Schizophrenia, psychosis, antipsychotics, drug development, mechanism of action

## Abstract

**Background:**

Schizophrenia is a disabling disorder that profoundly affects functioning and quality of life. While available antipsychotics have improved outcomes for patients with schizophrenia, they are relatively ineffective for negative and cognitive symptoms and are associated with a range of troublesome side effects. A significant unmet medical need for more effective and better-tolerated therapies remains.

**Methods:**

A roundtable consisting of 4 experts in the treatment of patients with schizophrenia convened to discuss the current treatment landscape, unmet needs from patient and societal perspectives, and the potential of emerging therapies with novel mechanisms of action (MOAs).

**Results:**

Key areas of unmet need include optimal implementation of available treatments, effective treatment of negative and cognitive symptoms, improvements in medication adherence, novel MOAs, avoidance of postsynaptic dopamine blockade–related adverse effects, and individualized approaches to treatment. With the possible exception of clozapine, all currently available antipsychotics primarily act by blocking dopamine D_2_ receptors. Agents with novel MOAs are urgently needed to effectively target the full range of symptoms in schizophrenia and facilitate an individualized treatment approach. Discussion focused on promising novel MOAs that have demonstrated potential in phase 2 and 3 trials include muscarinic receptor agonism, trace amine-associated receptor 1 agonism, serotonin receptor antagonism/inverse agonism, and glutamatergic modulation.

**Conclusions:**

Results from early clinical trials of agents with novel MOAs are encouraging, particularly for muscarinic and trace amine-associated receptor 1 agonists. These agents offer renewed hope for meaningful improvement in the management of patients with schizophrenia.

## INTRODUCTION

The introduction of antipsychotics in the 1950s transformed treatment of schizophrenia by providing effective therapy, primarily for positive symptoms (i.e., hallucinations, delusions), and allowing many patients to live outside the confines of the mental hospital. Unfortunately, currently available antipsychotics do not satisfactorily treat primary and persistent negative symptoms (Kantrowitz, 2017; Correll and Schooler, 2020) or cognitive deficits (Correll et al., 2023). Patients with schizophrenia have an estimated life expectancy 15–20 years less than the general population (Ringen et al., 2014; Correll et al., 2022c), with the metabolic and other side effects of antipsychotics being a potential contributor (Huhn et al., 2019; Meftah et al., 2020). The proportion of schizophrenia patients who have been “cured” has remained low over the last 70 years. Median recovery rates have remained stable or even decreased over time from before 1941 to after the introduction of atypical antipsychotics, with only 10%–20% of patients fully recovering (Jaaskelainen et al., 2013; Taylor and Jauhar, 2019; Huxley et al., 2021). Moreover, 10%–30% of patients with schizophrenia are refractory to antipsychotic drugs, and an additional 50%–60% derive only a partial response (Kane et al., 2019). Therapeutic nihilism reflects a growing pessimism regarding the ability to meet patient expectations and achieve treatment goals with currently available antipsychotics. Worsening of negative and cognitive symptoms may arise not only from medication side effects but also from illness progression. The resulting self-stigma, stigma from others, and lack of functionality leads to further functional limitations. Thus, effective treatments for negative and cognitive symptoms of schizophrenia are urgently needed to support patient functioning and hopefully prevent further deterioration.

The past 70 years of drug development for schizophrenia has focused on dopamine D_2_ functional antagonists, with all currently available antipsychotics (other than possibly clozapine) having this same core mechanism of action (MOA) (Kaar et al., 2020; Kantrowitz, 2021b). The multimodal effects of clozapine, including on glutamate, serotonin, α-adrenergic, muscarinic, and histamine receptors, may underlie its broad efficacy (de Bartolomeis et al., 2022). In addition, clozapine appears to act as a Trace Amine-Associated Receptor (TAAR1) agonist (Meyer and Stahl, 2019). The availability of an antipsychotic with similar efficacy as clozapine but with a better safety and tolerability profile would be a potential game changer. Additionally, drugs with novel MOAs may potentially treat the subgroup of patients whose symptoms are refractory even to clozapine or might help minimize the loss of efficacy by either supplementing the efficacy of other medications or otherwise modulating the response of the dopaminergic system during treatment.

Many recent attempts at developing drugs with novel MOAs have failed (Downing et al., 2014; Bugarski-Kirola et al., 2017), with results from animal models not translating to benefits in humans. Potential reasons for failure include increasingly high placebo response rates (Kemp et al., 2010), limitations in trial design or execution (Javitt and Kantrowitz, 2022), and, importantly, the lack of biomarkers to identify subpopulations whose neuropathology is likely to respond to the MOA of a drug (Kantrowitz, 2019; Stuke, 2023). However, this situation may be changing with numerous novel therapies with unique MOAs in late-stage clinical development (Correll et al., 2023). The development of novel antipsychotics targeting specific receptors may enable testing of theories about whether schizophrenia can arise primarily from dysfunction of specific neurotransmitter systems beyond dopamine (e.g., acetylcholine) (Schick et al., 2021), serotonin (Baltzersen et al., 2020), and glutamate (Kantrowitz and Javitt, 2009), in which case patients could possibly be matched to the agents most likely to address their particular pathophysiology, enabling personalized care and improved outcomes.

On March 16, 2022, a group of 4 experts in the treatment of patients with schizophrenia participated in a roundtable discussion, with a focus on the potential of emerging therapeutics with novel MoAs, focusing on investigational agents that have had at least 1 positive phase 2 study (Correll et al., 2022a). These included approaches targeting muscarinic acetylcholine receptors, TAARs, serotonin receptors, and glutamate modulation (Correll et al., 2022a) (Table 1). The proceedings of this roundtable discussion are summarized here as an update of novel MoAs in late-stage development in a format of a review of the public findings of these compounds. A key quote by participants is found in the inset. The discussion was sponsored by Karuna Therapeutics (Boston, MA, USA). Karuna did not influence the content and did not review the publication before submission.

**Table 1.** Selected Emerging Treatments for Schizophrenia^*a*^

Investigational agent	Company	Mechanism of action	Planned or ongoing phase 2 or 3 clinical trials in schizophrenia
Ulotaront (SEP-363856)	Sunovion/Sumitomo Pharma Co., Ltd.	TAAR1 agonist/5-HT_1A_ agonist	•NCT04825860 (phase 2/3): acute exacerbation •NCT04072354 (phase 3): acute exacerbation •NCT04092686 (phase 3): acute exacerbation •NCT04109950 (phase 3): long-term safety and tolerability •NCT04115319 (phase 3): long-term safety and tolerability vs quetiapine XR •NCT05359081 (phase 3): long-term safety and tolerability in Japanese patients
Ralmitaront (RO6889450)	Roche	TAAR1 partial agonist	•NCT03669640 (phase 2): schizophrenia or schizoaffective disorder and negative symptoms; monotherapy and adjunctive therapy phases
KarXT (xanomeline/trospium)	Karuna Therapeutics	M_1_/M_4_-preferring muscarinic receptor agonist/peripherally active anticholinergic	•NCT04738123 (phase 3): acute exacerbation •NCT04659174 (phase 3): long-term safety, tolerability, and efficacy (extension) study •NCT04820309 (phase 3): long-term safety, tolerability, and efficacy study •NCT05145413 (phase 3): adjunctive treatment in patients with inadequately controlled symptoms •NCT05304767 (phase 3): long-term safety and tolerability of adjunctive treatment in patients with inadequately controlled symptoms (extension study)
Emraclidine (CVL-231)	Cerevel	M_4_ muscarinic positive allosteric modulator	•NCT05227703 (phase 2): acute exacerbation •NCT05227690 (Phase 2): acute exacerbation
Pimavanserin	ACADIA Pharmaceuticals Inc.	Inverse agonist/antagonist at 5-HT_2A_ receptors with less potent action at 5-HT_2C_ receptors	•NCT04531982 (phase 3): adjunctive treatment for negative symptoms •NCT03121586 (phase 3): extension study to evaluate long-term safety and efficacy as adjunctive treatment
Roluperidone (Min-101)	Minerva Neurosciences	Antagonist at 5-HT_2A_ receptors and sigma-2 receptors	•N/A
Iclepertin (BI 425809)	Boehringer Ingelheim Pharma	GlyT1 inhibitor	•NCT03859973 (phase 2): added to current antipsychotic(s) and computer-based brain training for cognitive symptoms of schizophrenia •NCT04846868 (phase 3): add-on therapy for cognition and functional capacity •NCT04846881 (phase 3): add-on therapy for cognition and functional capacity •NCT04860830 (phase 3): add-on therapy for cognition and functional capacity •NCT05211947 (phase 3): long-term safety follow-up
Sodium benzoate (SND14)	SyneuRx International (Taiwan) Corp.	Unknown	•NCT02261519 (adaptive phase 2b/3): adjunctive treatment of schizophrenia •NCT01908192 (adaptive phase 2b/3): adjunctive treatment of adolescents with schizophrenia
Luvadaxistat (TAK-831)	Neurocrine Biosciences	DAAO inhibitor	•NCT05182476 (phase 2): add-on therapy for cognitive impairment

^*a*^Abbreviations: 5-HT, 5-hydroxytryptamine; DAAO, D-amino acid oxidase; GlyT1, glycine transporter 1; M, muscarinic; TAAR1, targeting trace amine-associated receptor 1.

(Dedic et al., 2019; Acadia Pharmaceuticals, 2020; S.K. Brannan et al., 2021b; Cerevel Therapeutics, 2021; Kantrowitz, 2021a; Pei et al., 2021; Correll et al., 2022a; Fleischhacker et al., 2021).

## Muscarinic Receptor Agonism

Xanomeline is a preferential M_1_/M_4_ muscarinic receptor agonist devoid of direct dopamine D_2_ receptor blocking activity (Shekhar et al., 2008). Stimulation of the M_4_ muscarinic auto-receptor in the brain reduces acetylcholine release from interneurons, leading to downstream effects, including a reduction in dopamine transmission (Threlfell et al., 2010; Correll et al., 2022a) and enhancement of glutamatergic neurotransmission (Dean and Scarr, 2020; Montani et al., 2021). The potential antipsychotic efficacy of xanomeline was serendipitously discovered during a clinical trial for Alzheimer’s dementia (Bodick et al., 1997), followed by a positive, small double-blind, placebo-controlled pilot study in schizophrenia (Shekhar et al., 2008). Further development of xanomeline was halted by treatment-limiting peripheral muscarinic M_1_ receptor agonist effects, particularly gastrointestinal (GI; e.g., nausea/vomiting) and syncope.

Recently, xanomeline was combined with a peripherally active anticholinergic drug (trospium chloride) that does not cross the blood-brain barrier. This combination, xanomeline/trospium chloride (KarXT; Karuna Therapeutics) was designed to reduce the frequency and severity of peripheral procholinergic side effects. Two trials of KarXT have been publicly reported (Brannan et al., 2021a; Correll et al., 2022b).

In a phase 2, multicenter, double-blind trial (NCT03697252), 182 patients with an acute exacerbation of schizophrenia were randomly assigned in a 1:1 ratio to receive 5 weeks of treatment with twice-daily KarXT (flexibly dosed and titrating up to 125 mg/30 mg twice daily) or placebo (Brannan et al., 2021a). The change from baseline to week 5 in the Positive and Negative Syndrome Scale (PANSS) total score was −17.4 points with KarXT compared with −5.9 points with placebo (least squares mean [LSM] difference of −11.6 points, *P* < .001; Cohen *d* effect size = 0.75). Most recently, in a publicly presented but not peer-reviewed first of 2 phase 3 studies, KarXT met its primary endpoint of a statistically significant reduction in PANSS total score vs placebo, with a *d* = 0.61 (Correll et al., 2022b). KarXT is also currently being evaluated in another phase 3 study (NCT04738123; Table 1) as well as in augmentation therapy in patients with schizophrenia with a suboptimal response to their current atypical antipsychotic treatment, except for olanzapine and quetiapine, which also have anticholingergic activity (NCT05145413; Table 1).

As expected, in the completed publicly available studies, the most common adverse events associated with KarXT were GI related—constipation (17%–21%), nausea (17%–19%), dry mouth (9%), dyspepsia (9%–19%), and vomiting (9%–14%)—but were generally mild and transient and did not lead to discontinuations (difference to placebo: 0%–1.5%) (Brannan et al., 2021a; Correll et al., 2022b). There were no adverse events related to extrapyramidal symptoms (EPS). The percentage of patients with ≥7% weight gain from baseline to week 5 was lower in the KarXT group than in the placebo group (2% vs 6% and 12.7% vs 15.2%), and no clinically relevant metabolic or endocrine abnormalities were observed (Brannan et al., 2021a; Correll et al., 2022b).

In addition to KarXT’s effect on total PANSS symptoms, KarXT also showed significant improvements vs placebo on PANSS positive and negative symptom scales. However, specificity of these effects is limited by the acutely exacerbated population and the possible confounds of improvement in secondary negative symptoms (Kantrowitz, 2017). Finally, a post hoc analysis of data suggests that KarXT may improve cognitive symptoms in the subgroup of patients with schizophrenia who have cognitive impairment at baseline (Sauder et al., 2021), although this benefit needs to be confirmed in a dedicated study of stable patients without significant positive symptoms.

A second muscarinic agent also recently reported results—a positive allosteric modulator that selectively acts on the M_4_ muscarinic receptor (CVL-231/emraclidine; Cerevel Therapeutics, Cambridge, MA, USA). Emraclidine demonstrated significant superiority vs placebo at both 20 b.i.d. and 30 mg qd in a 6-week, 81-participant, phase 1b trial in adult patients with acutely exacerbated schizophrenia (Krystal et al., 2023) and has proceeded to phase 2 development (NCT05227703, NCT05227690; Table 1). Compared with KarXT, emraclidine showed statistically similar effect sizes for the 20-mg (*d* = 0.59) and 30-mg (*d* = 0.68) groups for total PANSS vs placebo. Of note, the rates of GI side effects with emraclidine 20 mg b.i.d. (7%) and 30 mg qd (19%) were similar to placebo (15%), and the most common adverse events across doses were headache (26%–30%), nausea (7%), back pain (4%–7%), blood creatinine phosphokinase increase (4%–7%), dizziness (4%–7%), dry mouth (0%–11%), and somnolence (4%–7%). Similar to KarXT, these adverse events were generally mild and transient. There were no adverse events related to EPS. The percentage of patients with ≥7% weight gain from baseline to week 5 was lower in the emraclidine group than in the placebo group (14%–15%), and no clinically relevant metabolic or endocrine abnormalities were observed. Mild increases in systolic blood pressure (~1 mm Hg) and heart rate (4–5 beats per minute) were seen, and 6 participants had adverse events related to heart rate and blood pressure (3 in the placebo group and 3 in the emraclidine 20 mg b.i.d. group), but these were not considered clinically significant. Other muscarinic agents, including NBI-1117568, a M_4_ selective agonist (Neurocrine Biosciences, San Diego, CA, USA, NCT05545111), are currently in early-phase clinical development for schizophrenia.

An ongoing question regarding muscarinic agents is the relative advantages/disadvantages of functional agonism at M_1_ and M_4_. Activity at central M_1_ and M_4_ muscarinic receptors is believed to mediate antipsychotic effects, whereas agonism of central M_1_ muscarinic receptors is believed to additionally mediate potential beneficial cognitive effects (Dean and Scarr, 2020). Peripheral muscarinic M_1_ receptor agonist effects, however, appear to mediate GI adverse effects. Going forward, we speculate that agents with selective M_4_ functional agonism may exhibit relative GI tolerability advantages, while agents with M_1_ agonist activity may have additional cognitive enhancing advantages. Other considerations are frequency of dosing (qd vs b.i.d.).

## TAAR1 Agonism

Trace amines are endogenous amines present at low circulating levels (<100 ng/g tissue) (Berry et al., 2017). The TAAR1 is primarily located intracellularly but can transfer to the plasma membrane following heterodimerization with another receptor (Berry et al., 2017). In the presence of TAAR1 agonists, TAAR1 heterodimerizes with pre- and postsynaptic D_2_ receptors, resulting in internalization of receptors and thus reducing presynaptic dopamine synthesis and release via biased agonism of intracellular signaling pathways and D_2_ receptor membrane expression (Espinoza et al., 2011; Berry et al., 2017; Dedic et al., 2021). Although TAAR1-mediated functional antagonism of postsynaptic D_2_ receptors cannot be fully excluded, TAAR1 agonists do not directly antagonize postsynaptic dopamine D_2_ receptors (Leo et al., 2014; Kantrowitz, 2021b). By reducing presynaptic dopamine release, TAAR1 agonists might augment treatment with current antipsychotics by countering their effects on dopamine release, potentially allowing for lower doses of these medications. A role for glutamatergic modulation is also reported (Kantrowitz, 2021b). In addition, activation of peripheral TAAR1 receptors decreases appetite and increases satiety, delays gastric emptying, reduces fasting glucose in the liver, and modulates insulin production, raising the possibility that TAAR1 agonists may reduce the cardiometabolic burden associated with schizophrenia and antipsychotic treatment (Espinoza et al., 2011; Correll et al., 2022a).

The first TAAR1 agonist to reach clinical trials, SEP-363856 (ulotaront; Sunovion Pharmaceuticals, Marlborough, MA, USA; Sumitomo Pharma Co., Ltd., Osaka, Japan), was identified using SmartCube technology, an automated mouse behavioral platform that allows rapid screening of a large number of compounds to facilitate drug discovery (Roberds et al., 2011; Dedic et al., 2019). Ulotaront is also a partial agonist at serotonin 5-HT_1A_ receptors, suggesting potential antidepressant and antianxiety effects (Dedic et al., 2021). In a phase 2, multicenter, randomized, placebo-controlled trial (NCT02969382) of acutely exacerbated schizophrenia (Koblan et al., 2020), patients were randomized 1:1 to receive 4 weeks of once-daily treatment with ulotaront (at flexible doses of 50 mg or 75 mg; n = 120) or placebo (n = 125). Ulotaront significantly differentiated from placebo at 4 weeks on the PANSS total score (LSM change from baseline −17.2 vs −9.7, difference −7.5 points; *d* = 0.45, *P* = .001). Ulotaront was well tolerated, with an adverse event profile comparable with that of placebo. In a 6-month, open-label extension study that enrolled patients who completed the initial 4-week study, ulotaront treatment was associated with a mean (95% confidence interval) change from open-label baseline in the PANSS total score of −22.6 (Correll et al., 2021). Ulotaront treatment over 6 months was well tolerated and was associated with minimal changes in body weight and metabolic parameters and without clinically meaningful effects on serum prolactin levels or movement disorder scales. Multiple clinical trials are under way to further assess efficacy for acute exacerbations as well as long-term safety and tolerability (NCT04825860, NCT04072354, NCT04092686, NCT04109950, NCT04115319, NCT05359081; Table 1) (Hojlund and Correll, 2022).

The TAAR1 partial agonist ralmitaront (RO6889450; Roche, Basel, Switzerland) is recruiting for a phase 2 trial in patients with schizophrenia or schizoaffective disorder with negative symptoms (NCT03669640; Table 1), although a different phase 2 trial in patients with an acute exacerbation of schizophrenia or schizoaffective disorder was terminated early after it failed to meet the primary endpoint (change from baseline in PANSS total score, NCT04512066). An ongoing question on TAAR1 modulators is regarding relative advantages/disadvantages of partial vs full agonism. As recently reviewed, full agonists of TAAR1 appear to attenuate dopaminergic signaling, while partial agonists may potentially normalize or increase dopaminergic signaling (Liu et al., 2020). Behavioral studies of partial and full TAAR1 agonists, however, show overlapping results.

## Serotonin Receptor Antagonism/Inverse Agonism

Pimavanserin (ACADIA Pharmaceuticals Inc., San Diego, CA, USA) is a potent serotonin 5-HT_2A_ receptor inverse agonist (functional antagonist) and serotonin 5-HT_2C_ receptor antagonist that is approved for the treatment of Parkinson disease psychosis (Acadia Pharmaceuticals, 2020). Pimavanserin is believed to modulate dopaminergic activity through its functional serotonin 5-HT_2A_ receptor antagonism by indirectly decreasing cortical pyramidal glutamate cell firing, which decreases dopamine release by the ventral tegmental area in the mesolimbic pathway by indirectly increasing dopamine release in the mesocortical pathway as well as the nigrostriatal pathway that innervates the sensorimotor striatum (Kantrowitz, 2020; Stahl, 2021; Correll et al., 2022a). A phase 2, 6-month, multicenter, randomized, placebo-controlled trial (NCT02970305) evaluated adjunctive pimavanserin in stable outpatients with schizophrenia and predominant negative symptoms (Bugarski-Kirola et al., 2022). Patients received once-daily add-on therapy with flexibly dosed (10‒34 mg/d) pimavanserin (n = 201) or placebo (n = 202). Improvement from baseline to 6 months in the total 16-item Negative Symptom Assessment score (primary outcome) was significantly greater with pimavanserin (LSM, −10.4) vs placebo (LSM = −8.5, *P* = .043, *d* = 0.21); the effect size at the highest dose (34 mg) at final assessment was *d* = 0.34. Rates of treatment-emergent adverse events were similar between pimavanserin (40%) and placebo (35%) groups, and most were mild to moderate in severity. Pimavanserin showed no clinically relevant effects on weight, lipids, or glucose, although pimavanserin treatment produced greater changes compared with placebo in serum prolactin (mean change 24.7 vs −9.3) and creatinine kinase (mean change 29.4 vs −16.5). A phase 3 study (NCT04531982) is ongoing (Table 1).

Additionally, MIN-101 (roluperidone, Minerva Neurosciences, Burlington, MA, USA) is an investigational compound that is an antagonist at serotonin 5-HT_2A_ and sigma_2_ receptors. A monotherapy phase 2b study (Davidson et al., 2017) focusing on the PANSS negative factor score showed improvement for both the 32-mg and 64-mg groups compared with the placebo group (*P* ≤ .024, *d* = 0.45, and *P* ≤ .004, *d* = 0.57, respectively). The phase 3 follow-up (NCT03397134) failed to meet its prespecified primary outcome but did show a trend-level significance favoring roluperidone monotherapy on the primary endpoint (PANSS negative factor score, *P* = .06) at 12 weeks (Davidson et al., 2022) (Table 1).

## Glutamatergic Modulation

The rationale for treatment strategies targeting glutamate transmission is supported by data implicating dysfunction of the glutamatergic system in the pathophysiology of schizophrenia (Kantrowitz and Javitt, 2009). Glutamate is an excitatory neurotransmitter widespread throughout the brain and acts on ionotropic and metabotropic glutamate receptors. Early evidence for the role of the *N*-methyl-d-aspartate receptor (NMDAR) in schizophrenia is derived from animal and human studies, showing that administration of NMDA antagonists, such as ketamine and phencyclidine, reproduces the cardinal symptoms of schizophrenia, including not only positive symptoms but also chronic negative symptoms and cognitive dysfunction (Javitt and Zukin, 1991).

Glycine is a necessary co-agonist of the NMDAR (Kleckner and Dingledine, 1988), and various strategies have sought to enhance NMDA function via direct administration of glycine or similarly acting co-agonists (d-serine) or by preventing breakdown or reuptake of glycine or D-serine (Javitt and Kantrowitz, 2022). Studies of glycine agonists have yielded inconsistent results and have not led to US Food and Drug Administration–approved treatments (Javitt and Kantrowitz, 2022). A phase 2 study (NCT00616798) of augmentation therapy with bitopertin, a high-affinity glycine transporter type I inhibitor that blocks glycine reuptake showed benefit for negative symptoms in a per-protocol population (Umbricht et al., 2014); however, a subsequent phase 3 study enrolling patients with predominant negative symptoms failed to demonstrate a significant benefit (Bugarski-Kirola et al., 2017). A phase 2, randomized, double-blind, placebo-controlled study (NCT02832037) of add-on therapy with the potent and selective glycine transporter type I inhibitor BI 425809 (iclepertin, Boehringer Ingelheim Pharma, Biberach, Germany) demonstrated significant (*d* = 0.34) improvements in cognition over 12 weeks of treatment in patients with schizophrenia (Fleischhacker et al., 2021). A phase 2 study (NCT03859973; Table 1) is evaluating iclepertin added to current antipsychotic therapy and computer-based training for cognitive symptoms of schizophrenia, and phase 3 studies of add-on therapy with iclepertin are underway (NCT04846868, NCT04846881, NCT04860830, NCT05211947; Table 1).

d-Serine is metabolized by d-amino acid oxidase (dAAO). Studies have demonstrated reduced d-serine levels in the plasma and cerebrospinal fluid as well as elevated dAAO activity in the cortex of patients with schizophrenia (Madeira et al., 2008; Sacchi et al., 2013). These findings have led to the hypothesis that excessive dAAO activity leads to reduced d-serine levels, which may then contribute to NMDAR hypofunction in schizophrenia (Meftah et al., 2021). dAAO is primarily active in the cerebellum, small intestine, liver, and kidney, and it is hypothesized that a dAAO inhibitor may be beneficial in schizophrenia via reduced peripheral degradation of d-serine rather than by direct cortical activity.

Sodium benzoate (SyneuRx International Corp, Taiwan) was initially studied as a dAAO inhibitor, but it does not appear to impact d-serine levels in vivo, and the exact mechanism of action is unclear (Huang et al., 2023). Clinical trials evaluating sodium benzoate suggest possible benefits (Lane et al., 2013; Lin et al., 2018), and phase 2/3 studies are recruiting (NCT02261519, NCT01908192; Table 1). In a phase 2 study, add-on therapy with another dAAO inhibitor (TAK-831/luvadaxistat; Neurocrine Biosciences Inc.) was not effective for negative symptoms of schizophrenia but showed a signal for improving cognitive symptoms (Murthy et al., 2021), and a phase 2 study in patients with cognitive impairment associated with schizophrenia (NCT05182476) is underway (Table 1).

While high-profile failures using drugs targeting glutamate transmission, such as bitopertin and pomaglumetad (Downing et al., 2014; Bugarski-Kirola et al., 2017), have challenged the validity of this treatment approach, it is unclear whether the failures were due in part to inadequate dosing (Kantrowitz et al., 2017), suboptimal dosing intervals, or imprecise patient selection. This situation argues for the use of prespecified validated pharmacodynamic “target engagement” biomarkers early in drug development to assess whether a larger clinical trial is warranted, and if so, the optimal dose to be used (Kantrowitz et al., 2020; Sehatpour et al., 2023).

## CONCLUSIONS

New therapies in development that do not directly target dopamine receptors and have demonstrated robust efficacy in phase 2 and 3 clinical trials, with signals of efficacy for negative and possibly also cognitive symptoms, represent a potential paradigm shift in the treatment of schizophrenia. These agents appear well tolerated and thus far not associated with the EPS, weight gain, sedation, sexual dysfunction, and metabolic effects that have limited the use of currently available antipsychotics (Solmi et al., 2017). If phase 3 studies continue to confirm the results observed in phase 2 studies, leading to regulatory approval, these novel compounds will offer renewed hope for meaningful improvement and may provide psychiatrists with the tools they need to more confidently switch or augment antipsychotic medication (when appropriate) to address the full constellation of patients’ symptoms and effectively treat a larger group of patients.

Although this speculation goes beyond the available data, it is possible antipsychotics with novel MOAs may have pleomorphic effects—for example, they may offer disease modification that may translate into preventing relapses. If subsets of patients with treatment-resistant schizophrenia have a pathology primarily characterized by nondopaminergic dysfunction, then novel agents may be able to successfully treat some of these patients, including possibly even those whose symptoms are resistant to clozapine. The availability of agents with different MOAs may allow fine-tuning of medications to treat symptom domains and target multiple pathways with treatment combinations, possibly producing a synergistic effect. Additionally, many patients who are otherwise doing well on their antipsychotic therapy may consider discontinuing treatment due to weight gain or other side effects, and the addition of an agent with a novel MOA might enable dose reduction of current medication, resulting in improved tolerability.

## Interest Statement

Dr Kantrowitz reports having received consulting payments within the last 24 months from Alphasights, Medscape, Putnam, techspert.io, Health Monitor, Third Bridge, MEDACorp, Trinity, Globaldata, GKA, Clearview, Clarivate, Health Advances, ECRI Institute, ExpertConnect, Acsel Health, Slingshot, Antheum, Guidepoint, SmartAnalyst, First Thought, Wedbush, Jefferies, Otsuka, Vox Neuro, and Reckner. He has served on the MedinCell Psychiatry, Tolmar, Merck, Leal, and the Karuna Advisory Boards. He has conducted clinical research supported by the NIMH, Sunovion, Roche, Cerevance, Click, Neurocrine, Corcept, Taisho and Boehringer Ingelheim within the last 24 months. He owns a small number of shares of common stock from GSK. Dr Correll has been a consultant and/or advisor to or has received honoraria from AbbVie, Acadia, Alkermes, Allergan, Angelini, Aristo, Boehringer Ingelheim, Cardio Diagnostics, Cerevel, CNX Therapeutics, Compass Pathways, Darnitsa, Gedeon Richter, Hikma, Holmusk, Intra-Cellular Therapies, Janssen/Johnson & Johnson, Karuna Therapeutics, LB Pharma, Lundbeck, MedAvante-ProPhase, MedinCell, Merck, Mindpax, Mitsubishi Tanabe Pharma, Mylan, Neurocrine Biosciences, Newron, Noven, Otsuka, Pharmabrain, PPD, Recordati, Relmada, Reviva, ROVI, Seqirus, SK Life Science, Sunovion, Sun Pharma, Supernus, Takeda, Teva, and Viatris; has provided expert testimony for Janssen and Otsuka; has served on a data safety monitoring board for Lundbeck, Relmada, Reviva, ROVI, Supernus, and Teva; has received grant support from Janssen and Takeda; has received royalties from UpToDate; and is a stock option holder of Cardio Diagnostics, Mindpax, LB Pharma, and Quantic. Dr. Jain reports AbbVie (Allergan): Consultant, research, paid speaker; Acadia: Consultant; Adamas: Consultant, advisory board; Alfasigma: Consultant; Alkermes: Paid speaker, advisory board; Axsome: Consultant, paid speaker; Biogen: Consultant; Boehringer Ingleheim: Consultant; Cingulate Therapeutics: Consultant; Corium: Consultant, paid speaker, advisory board; Eisai: Consultant paid speaker, advisory board; Evidera: Consultant; Impel: Consultant; Intra-Cellular Therapies: Paid speaker; Ironshore Pharmaceuticals: Paid speaker; Janssen: Consultant, paid speaker, advisory board; Lilly: Consultant, research, paid speaker, advisory board; Lundbeck: Consultant, research, paid speaker, advisory board; Merck: Consultant, paid speaker, advisory board; Neos Therapeutics: Consultant, paid speaker, advisory board; Neurocrine Biosciences: Consultant, advisory board; Osmotica: Consultant; Otsuka: Consultant, research, paid speaker, advisory board; Pamlab: Consultant, paid speaker, advisory board; Pfizer: Consultant, research, paid speaker, advisory board; Sage Therapeutics: Consultant, advisory board; Shire: Consultant, research, paid speaker, advisory board; Sunovion: Consultant, paid speaker, advisory board; Supernus: Consultant, advisory board; Takeda: Consultant, research, paid speaker, advisory board; Teva: Consultant, advisory board; Transcend Therapeutics: Consultant; Tris Pharmaceuticals: Paid speaker; Usona: Advisory board. Dr. Culter reports Consultant: AbbVie (Allergan), Acadia, Alfasigma, Alkermes, Axsome, Biogen, BioXcel, Boehringer Ingelheim, Cerevel, Corium, Gedeon Richter, Idorsia, Intra-Cellular Therapies, Ironshore Pharmaceuticals, Janssen, Jazz Pharmaceuticals, Karuna, Lundbeck, Luye Pharma, Neumora, Neurocrine, Noven, Otsuka, Relmada, Reviva Pharmaceuticals, Sage Therapeutics, Sunovion, Supernus, Takeda, Teva, Tris Pharma, VistaGen Therapeutics; Speaker/Promotional Honoraria: AbbVie (Allergan), Acadia, Alfasigma, Alkermes, Axsome, BioXcel, Corium, Idorsia, Intra-Cellular Therapies, Ironshore Pharmaceuticals, Janssen, Lundbeck, Neurocrine, Noven, Otsuka, Sunovion, Supernus, Takeda, Teva, Tris Pharma; Data Safety Monitoring Board (DSMB): COMPASS Pathways; Employee & Board Member: Neuroscience Education Institute.

## LOOKING FORWARD—EXPERT OPINION

### How do you see the field of schizophrenia evolving in the years ahead? What predictions do you have for 20 years in the future?

*Dr. Correll*: In the next 20 years, and maybe even in the next 10 years, we will have more precision medicine, which will come from our ability to bring medications with new MOAs into clinical use. We will have more opportunities to stratify patients for treatment based on pharmacological challenge tests, greater personalization of treatment, and more effective prevention of clinical worsening. If we can identify and treat people so that they do not lose as much brain potential and surrounding psychosocial fabric, then this may lead to more enduring success.

*Dr. Kantrowitz*: We will be able to use biomarkers to potentially narrow down which treatment or MOA might be right for an individual patient. For example, we could use electrophysiological or imaging biomarkers that are suggestive of deficits and potentially enhanced by glutamatergic medications; if we could give someone a challenge with a glutamatergic medication and see if the electrophysiological signal gets better, then this may suggest that the patient could benefit from the glutamatergic medication.

*Dr. Jain*: We may have phenotypic markers—is your patient doing well? What symptoms or side effects remain that are causing their burden? Those phenotypic biomarkers are available right now, and that is where I get excited. I really hope personalized medicine will be a reality the way it has become in oncology and infectious disease, among other areas. New MOAs bring the possibility that, even if the scientific advances in the understanding of the disease are still lagging, clinicians can do better tomorrow than they are doing today. We can address more symptoms, we can improve tolerability, and maybe we can even provide some disease modification.

*Dr. Cutler*: We are likely to have 2 new categories of medications, muscarinic receptor agonists and TAAR1 antagonists, in the very near term. These would provide new tools with new MOAs that may position clinicians to do better for a broader range of patients. We may have tools to select the appropriate medications for the appropriate patients, whether that is using what we know about the data from trials showing how medications may work better for a certain kind of clinical cluster or what we know about the MOA or AE tolerability profile.
